# Reliability modeling of the fatigue life of lead-free solder joints at different testing temperatures and load levels using the Arrhenius model

**DOI:** 10.1038/s41598-023-29636-3

**Published:** 2023-02-13

**Authors:** Dania Bani Hani, Raed Al Athamneh, Mohammed Abueed, Sa’d Hamasha

**Affiliations:** 1 Hijjawi Faculty for Engineering Technology, Yarmouk University, Irbid, 21163 Jordan; 2 Department of Industrial Engineering, Faculty of Engineering, The Hashemite University, P.O. BOX 330127, Zarqa, 13133 Jordan; 3 Intel Corporation, 2775 NE John Olsen Ave, Apt H116, Hillsboro, 97124 USA; 4 Department of Industrial and Systems Engineering Samuel Ginn College of Engineering, Auburn University, Auburn, AL 36849 USA

**Keywords:** Electrical and electronic engineering, Mechanical engineering, Electronic devices, Materials for devices

## Abstract

Reliability of the microelectronic interconnection materials for electronic packages has a significant impact on the fatigue properties of the electronic assemblies. This is due to the correlation between solder joints reliability and the most frequent failure modes seen in electronic devices. Due to their superior mechanical and fatigue properties, SAC alloys have supplanted Pb-solder alloys as one of the most commonly used solder materials used as interconnection joints on electronic packages. The main aim of this study is to develop a prediction model of the fatigue life of the solder joints as a function of the experimental conditions. Using a customized experimental setup, an accelerated fatigue shear test is applied to examine the fatigue life of the individual SAC305 solder joints at actual setting conditions. OSP surface finish and solder mask defined are used in the studied test vehicle. The fatigue test includes three levels of stress amplitude and four levels of testing temperature. A two-parameter Weibull distribution is used for the reliability analysis for the fatigue life of the solder joints. A stress–strain curve is plotted for each cycle to construct the hysteresis loop at each cyclic load and testing temperature. The acquired hysteresis loop is used to estimate the inelastic work per cycle and plastic strain. The Morrow energy and Coffin Manson models are employed to describe the effects of the fatigue properties on the fatigue life of the solder joints. The Arrhenius model is implemented to illustrate the evolutions in the stress life, Morrow, and Coffin Manson equations at various testing temperatures. The fatigue life of SAC305 solder joints is then predicted using a general reliability model as a function of the stress amplitude and testing temperature.

## Introduction

The fatigue life of the microelectronics interconnection materials is a major indicator of the reliability of the electronic assemblies, as a single failure in these connections could result in the destruction of the entire electronic system or a drastic reduction in its operational performance. Solder joints and other interconnection materials are fundamentally subject to various types of thermal and mechanical stresses in real-life applications such as shear, tensile, creep, mechanical and thermal shock, and fatigue stresses^1–4^. The thermal cycling phenomenon, which is commonly observed in harsh environmental conditions, is one of the major sources of the combined thermal and mechanical stresses. The fatigue shear stress induced by the thermal cycling phenomenon has a substantial impact on the fatigue life of the solder joints. The mismatch between the coefficient of thermal expansion (CTE) of the printed circuit board (PCB), the solder joints, and the electronic package is the primary cause for having the fatigue shear stress of the solder joints^5,6^.In contrast, solder joints are immediately subjected to thermal stress during the thermal cycling process. As a result of the applied elevated temperatures, the aging effect will impact the performance of the solder joints. Aging is another factor that influences fatigue life degradation. The effects of aging on the fatigue life behavior of the solders are strongly dependent on the temperature and exposure time^7–9^. 

In this study, the effect of the thermal cycling process on the electronic packages was examined by applying an accelerated fatigue shear test that considered individual solder joints at different testing temperatures. Several studies investigated the mechanical and fatigue behavior of different solder alloys. Basit et al. developed a new prediction methodology for the reliability of SAC alloys by conducting a thermal cycling accelerated life test for the pre-aged microelectronic interconnection materials and finite element analysis. Energy dissipation per life cycle and the Anand viscoplastic model were used to estimate the fatigue life of the SAC305 solder joints through the thermal cycling test. In their investigation, four levels of aging temperature and three levels of aging time were utilized. The thermal cycling process was utilized following the aging process of the electronic packages, with cycling temperatures ranging from − 40 to 125 °C. The effects of aging temperature and time on the Anand model were found. The modified Anand model in conjunction with the finite element model were utilized to predict the stress strain histories of SAC305 solder joints. The simulation results were compared with Weibull reliability analysis for real experimental data to validate the new prediction approach^10^. Chen et al. studied the mechanical and thermal reliability behaviors of SAC305 and SAC-Sb using thermal analysis. In the study, two different levels of the operating temperature and strain rate were considered for analyzing the mechanical behavior. The Anand model was utilized to examine the fatigue thermal resistance of the studied solder alloys. Employing SAC-Sb solder joints resulted in a significant degradation in the inelastic strain. Moreover, SAC-Sb solder joints demonstrated a significant fatigue resistance in harsh operating environments^11^. The thermomechanical lifetime of the solder joint was examined by Jiao et al. under electric current effects in temperature cycling conditions. Sn3.8Ag–0.5Cu solder paste with two solder ball types (barrel and hourglass) and different current densities was utilized. Finite element analysis was performed to simulate the effect of the combined thermal cycling and electric current on the thermomechanical lifetime. Under the stated experimental conditions, the hourglass solder joint type showed a lower fatigue lifetime compared with the barrel solder joint^12^. Samavatian et al. explored the influence of random frequency vibration on the fatigue life of the solder joints. The study utilized ball grid array in three different circuit boards as a test vehicle. The finite element method was used to identify the best circuit board configuration in terms of fatigue life. The effect of the input frequency is measured by applying an acceleration power spectral density and the failures were defined based on the value of the root squared peeling stress. According to the results of the finite element analysis, the solder joints located in the corners of the BGA were more susceptible to failure. Furthermore, compared to the other board designs, the board configuration with a heat sink in corners of the board demonstrated a high fatigue resistance performance^13^.

Furthermore, effects of aging on the fatigue life and mechanical properties were examined by various studies using different types of the mechanical and thermal accelerated tests. The effect of aging time on the fatigue life of two different solder alloys (SAC305 and SAC305+Bi) was demonstrated by Al Athamneh et al. . An accelerated fatigue shear test was employed to test the individual solder joints using a customized experimental setup. Three different stress amplitude values were applied at different levels of the aging time. A value of 100 °C for aging temperature was utilized in the study. The SAC305+Bi solder joints exhibited a high level of performance in terms of the number of cycles to failure and the rate of degradation in the fatigue life when compared with the SAC305 solder joints. In addition, a slight improvement in the fatigue life of the SAC305+Bi solder joints was achieved in the first hours of aging, and the fatigue life started decreasing after 10 h of aging^14^. In another study implemented by Bani Hani et al., the effect of aging temperature on the fatigue life of SAC305 solder joints was investigated. The accelerated fatigue shear test was utilized to demonstrate the fatigue life of the individual solder joints at different stress amplitudes and aging temperature values. All examined solder joints were aged for 100 h, and the obtained fatigue life results were compared with the non-aged solder joints for the same stress amplitude levels. A significant degradation in the fatigue life was observed when either the stress amplitude or aging temperature were increased. The Arrhenius equation was utilized to build a reliability model as a function of testing temperature and fatigue properties^15^. Roumanille et al. exhibited the fatigue life of ball grid array with lead-free solder joints under different aging conditions. The results indicated that increasing the aging temperature leads to a growth in the precipitates sizes due to the coarsening of the precipitates. The Weibull distribution was used in the failure analysis. A reduction in the fatigue life was observed when the electronic packages are aged at elevated temperatures. When the aging temperature was increased above 100 °C, the degradation rate in the fatigue life was significantly reduced^16^.

Some previous studies also investigated the effect of testing temperature on SAC solder alloys reliability using different accelerated tests and experimental conditions. For example, the impact of different levels of testing temperature on the cyclic stress strain behavior was investigated by Haq et al. Two solder alloys (SAC305 and SAC-Q) with uniaxial testing specimens form were utilized in the experiment. Two levels of aging were applied for the studied alloys and the obtained results were compared with the non-aged solder joints. The levels of the studied testing temperatures were between 25 and 100 °C. The evolutions in the hysteresis loop, peak stress plastic strain and inelastic work were determined for both solder alloys at different aging and testing temperatures. The solder joints that were tested at elevated temperatures showed a significant reduction in loop area and peak stress. In contrast, the plastic strain range was directly proportional to the increase in the testing temperature. The peak stress and loop area for SAC305 alloy were lower than those of SAC-Q solder alloy. Negative impact of aging was observed on the mechanical fatigue properties for both solder alloys^17^. Lall et al. investigated the impacts of the low testing temperatures on the mechanical properties of SAC305 and SAC105 solder alloys. Different levels of the strain rate and aging temperature were utilized as another testing parameter in their experiment. The ultimate tensile strength, yield strength, and elastic modulus were used to describe the mechanical behavior of the studied solder alloys. The results indicated that the impact of changing the levels of the testing temperature on the mechanical properties was higher than the effect of increasing the aging level for both solder alloys. The Anand models were constructed based on the obtained stress strain data for each alloy, and the experimental data were employed to validate the Anand model^18^.

According to the discussed literature, developing a systematic method for the reliability modeling of the solder joints under different operating temperatures is a problematic subject. Therefore, a systematic approach was proposed in this study to estimate the reliability distribution of the individual SAC305 solder joints under actual setting conditions as a function of the different operating conditions and fatigue properties. In this study, an accelerated fatigue shear test is used to examine the fatigue life of the individual SAC305 solder joints at actual setting conditions. Three levels of stress amplitude and four levels of testing temperature were used as experimental conditions. A Two- parameter Weibull distribution was employed to perform the fatigue failure analysis. A general reliability model was constructed using the stress life and Arrhenius equations to predict the solder joints’ reliability as a function of stress amplitude and testing temperature. The Arrhenius equation was utilized as well to develop two other reliability models using the Coffin Manson and Morrow energy models.

## Materials and methods

**Figure 1:**
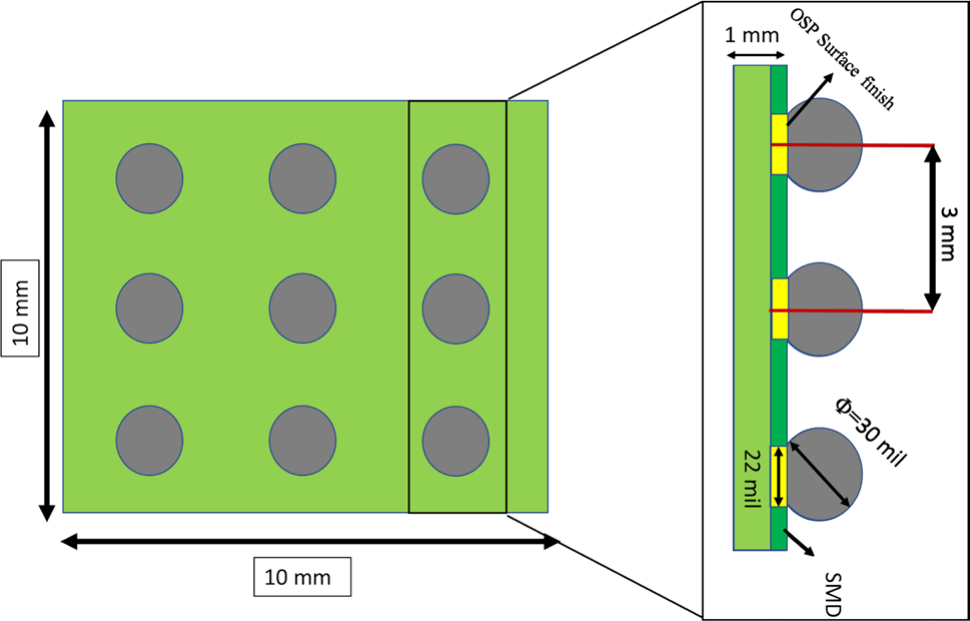
The test vehicle.

**Figure 2:**
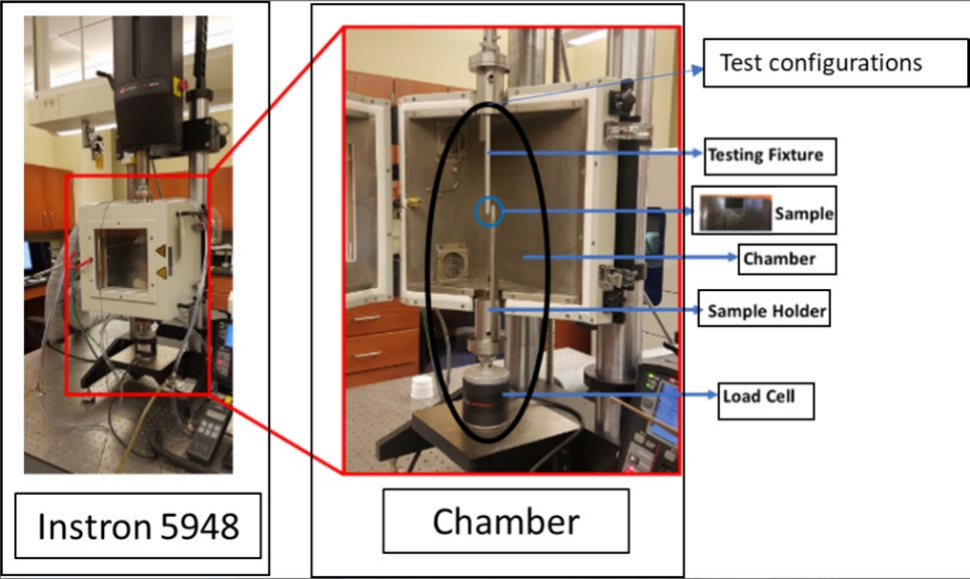
Intron testing machine and test configurations.

In this study, an array of SAC305 (Sn 96.5%–Ag 3%–Cu 0.5%) solder joints that were installed in the customized circuit board was utilized as a test vehicle. FR-4 epoxy glass fabric composite and SAC305 solder alloy were used to fabricate the printed circuit board (PCB) and the solder joints in the studied test vehicle, respectively. Two types of stencils with different diameters were utilized in the test vehicle preparation. The first stencil that has a small diameter (22 mil) was implemented to print sticky flux in the PCB. On the other hand, the large stencil was employed to apply the solder joints on the surface of the installed flux, where the outer and inner aperture diameters for the used stencil were 60 mil and 30 mil, respectively. A reflow oven with ten zones, controlled temperature, and nitrogen environment was used in the surface mounting process for the test vehicle. Surface Mask Define (SMD) and OSP surface finish were utilized in PCB manufacturing. Figure 1 depicts the utilized test vehicle. The solder ball and copper pad diameters were 30 mil and 22 mil, respectively. The pitch distance between the adjacent solder joints was 3 mm. An Instron micro tester machine that is attached with a customized chamber is used to conduct the accelerated fatigue shear test at different stress and testing temperature levels. A special fixture was designed and manufactured to adapt the individual solder joints in the test vehicle to the testing machine configuration. Figure 2 represents the Intron testing machine and the experimental setup configurations. The chamber shown in Fig. 2 was used to control the testing environmental temperature. Four levels of the testing temperature (− 10 °C, 25 °C, 60 °C and 100 °C) and three levels of the load levels ( 16 MPA, 20 MPa and 24 MPa) were used as experimental parameters. The stress amplitude levels were determined based on the opportunity to obtain a reasonable fatigue life cycle. Due to the stochastic nature of the fatigue life, the reliability analysis of the short fatigue life might lead to incorrect conclusions about the fatigue resistance, and the accuracy of the obtained reliability prediction model. Furthermore, it can produce misleading results in terms of the factors contributions to solder joints fatigue behavior. Moreover, the long fatigue life cycle requires more experimental time and large computational capacity to handle and process the generated data . Several experiments were conducted at different conditions to determine the appropriate load levels that could provide a reasonable number of fatigue life cycles. The testing temperature levels were defined based on the common range of the operating temperatures of the electronic components in harsh environmental conditions. The used shear strain rate for cycling the solder joints was 0.1 s^−1^. Regarding the full factorial matrices for the experiments design, L_12,_ orthogonal array shown in Table 1 was used as a test matrix for the study^19^. After performing some of the experimental tests and acquiring consistent fatigue life data at different conditions, seven replicates were utilized as the data points to represent the fatigue behavior of the solder joints at each experiment.

**Table 1:** The proposed test matrix.

Stress amplitude (MPa)	Testing temperature (°C)	Number of replicates
16	− 10	7
	25	7
	60	7
	100	7
20	− 10	7
	25	7
	60	7
	100	7
24	− 10	7
	25	7
	60	7
	100	7

In order to analyze the fatigue failure of the solder joints, two parameters Weibull distribution was used to identify the reliability model of the solder joints at each experimental condition. The least squares method was applied for parameters estimation of the Weibull distribution. Equation (1) shows the two parameter Weibull model equation, where the scale and shape parameters are denoted by symbols *β* and *α*, respectively. The shape parameter represents the slop of the probability plot of the Weibull distribution and the number of cycles at which 63.2% of the population from the studied components has failed^20,21^. After extracting the reliability model for each experimental condition, the stress life equation indicated in Eq. (2) is used to develop a prediction model of the characteristic life (scale parameter) at different values of the stress amplitude^22,23^. Where the characteristic life and the stress amplitude are represented by *N*_*63*_ and *P*, respectively. *Q* and *c* are the material constants for which the ductility index can be described by the constant *c*. High values of the constant *c* displayed a strong indication of the low ductility of the material. The impact of changing the testing temperature on the stress life equation was illustrated by using the Arrhenius equation provided in Eq. (3)^24,25^. Where *A* and *B* represent the Arrhenius equation constants, *T* is the testing temperature (Kelvin), and *r* is the process rate. The Arrhenius equation was employed to develop a model to estimate the stress life equation constants at different testing temperatures. The obtained Arrhenius prediction equations for the equation constant and the stress life equation were used to have a prediction model of the characteristic life. The obtained prediction equation for the fatigue life is substituted in the Weibull reliability model instead of the scale parameter. If there is no apparent behavior observed for the shape parameter when either the stress amplitude or testing temperature are changed, the mathematical average for all the shape parameter values is calculated and used in the Weibull reliability model.

1 $$R\left( t \right) = e^{{ - \left( {\frac{t}{\alpha }} \right)^{\beta } }}$$

2 $$N_{63} = Q*P^{ - c}$$

3 $$r = A*e^{{ - \frac{B}{T}}}$$

Two other solder joint reliability models can be developed from the fatigue properties of the solder joints. First, the hysteresis loop for each cycle from each replicate should be constructed using the stress strain curve. The area inside the hysteresis loop represents the inelastic work, and the strain at zero stress is denoted by the plastic strain. In order to calculate the averages of inelastic work and plastic strain for each replicate, the three regions of fatigue life of the solder joints should be identified by plotting the inelastic work or the plastic strain values versus the life cycles. The regions obtained are the strain hardening, steady state, and crack growth. The averages of the inelastic work and plastic strain for each replicate were determined at the steady state region. Then, the averages of the inelastic work and plastic strain per cycle were calculated at each experimental condition in the steady state region. The Morrow energy and Coffin Manson models were utilized to illustrate the relationships between the characteristic life, average inelastic work per cycle, and average plastic strain per cycle. The Morrow energy model shown in Eq. (4) is a power equation that was employed to illustrate the relationship between the inelastic work and the fatigue life, or the characteristic life. Where *Z* ( the fatigue exponent) and *R* (the ductility coefficient) are the equation constants, and *W* is the average inelastic work per cycle^26,27^. In addition, the plastic stain was employed to model the fatigue life using a power equation provided in Eq. (5). The average plastic strain per cycle was symbolized by *PS*. The fatigue exponent and the ductility coefficient are denoted by *M* and *U*, respectively^28,29^.

4 $$N_{63} = R^{\frac{1}{Z}} W^{{\frac{ - 1}{Z}}}$$

5 $$N_{63} = U^{\frac{1}{M}} PS^{{\frac{ - 1}{M}}}$$

Thus, the Arrhenius equation, stress life equation, Coffin Manson model and Morrow energy model were utilized in this study to construct three reliability models of SAC305 solder joints at different fatigue properties, working temperatures and load levels. The original data in this study was utilized before to model SAC305 solder joints fatigue life using different modeling methodologies and tools (Fuzzy inference system)^30^, at which the proposed prediction models in the current study enhanced the predictability, simplicity, and accuracy of fatigue life modeling for SAC305 solder joints at different working temperatures and stress amplitudes.

## Results and discussion

### Fatigue failure analysis using weibull distribution

**Figure 3:**
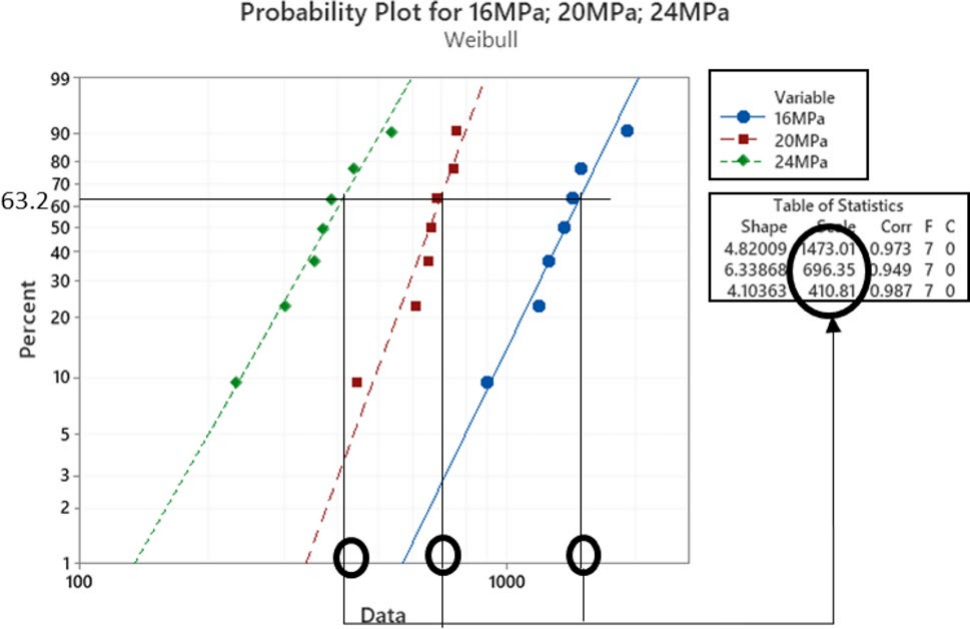
The Weibull probability plots for SAC305 solder joints cycled at room temperature and at different load levels (reused and modified from)^30^.

**Figure 4:**
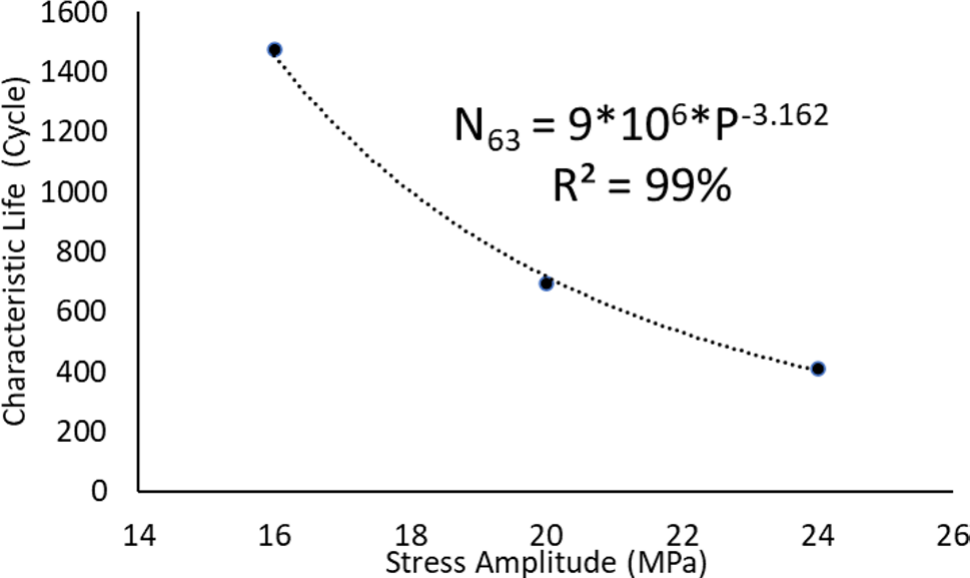
The Stress life equation for SAC305 solder joints that were cycled at room temperature.

**Figure 5:**
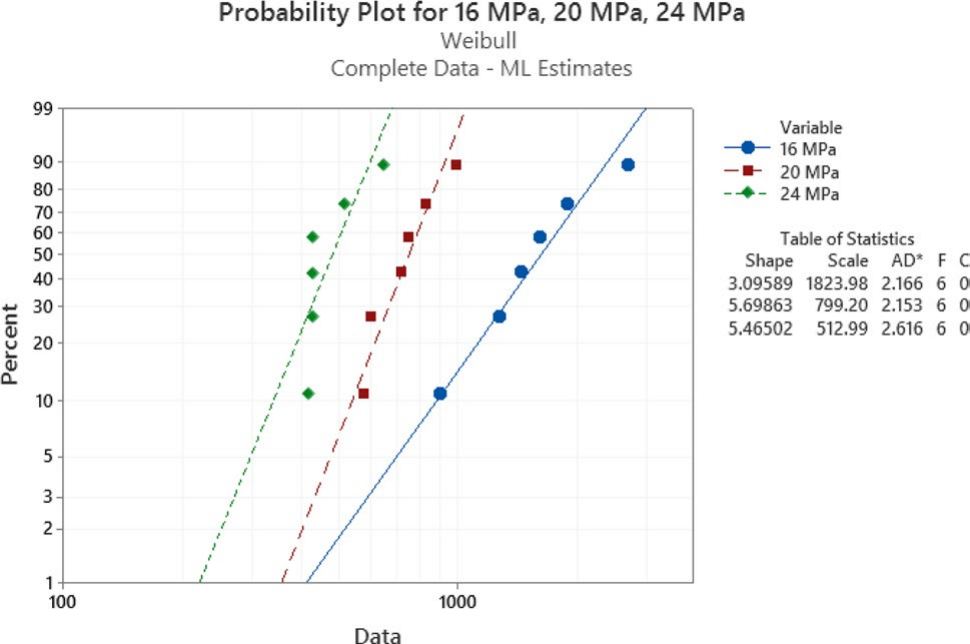
The Weibull probability plots for SAC305 solder joints cycled at − 10 °C and different load levels.

**Figure 6:**
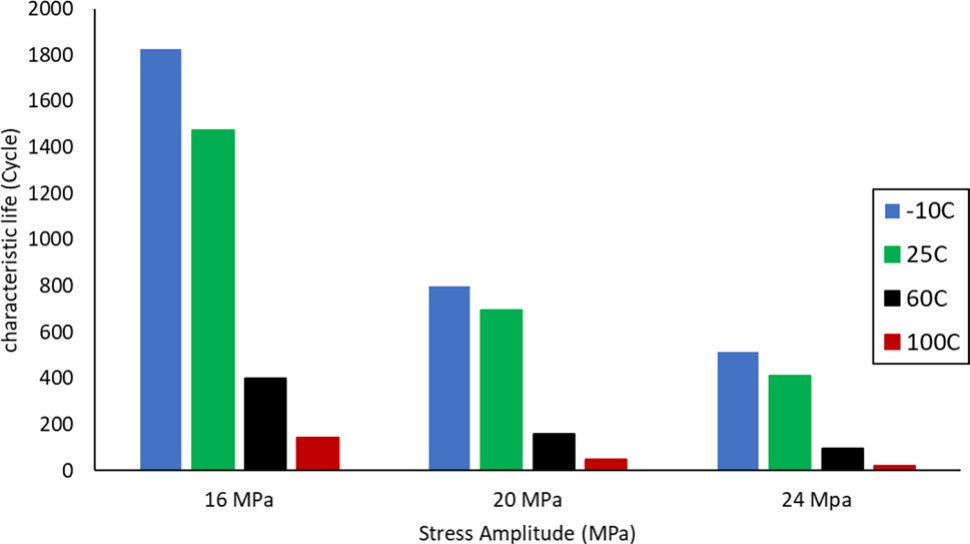
Bar chart for the degradations in the characteristic life when the stress amplitude or the testing temperature are increased.

**Figure 7:**
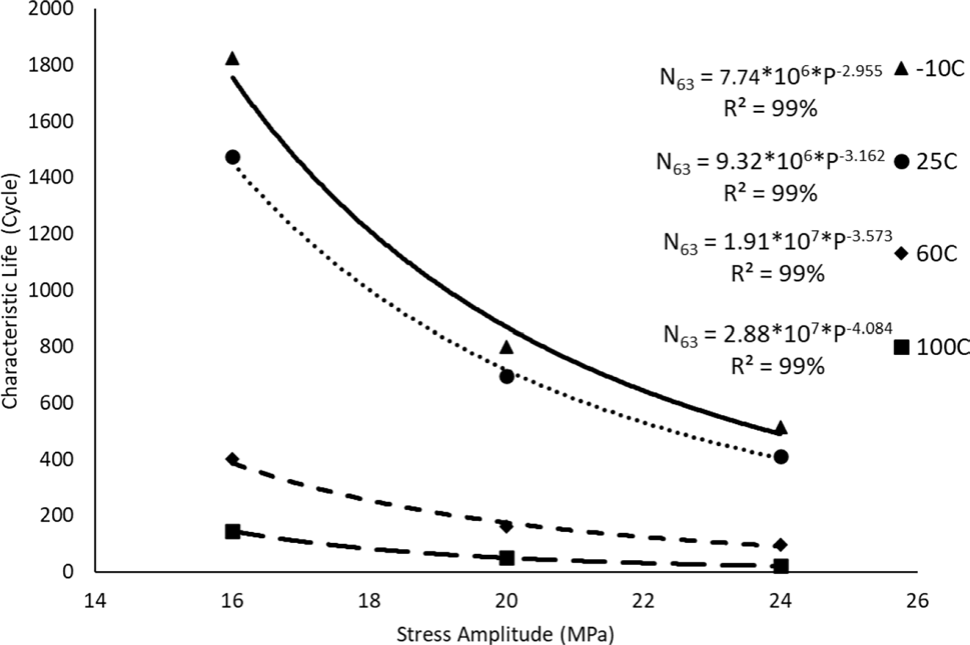
The stress-life equation at different testing temperatures.

**Figure 8:**
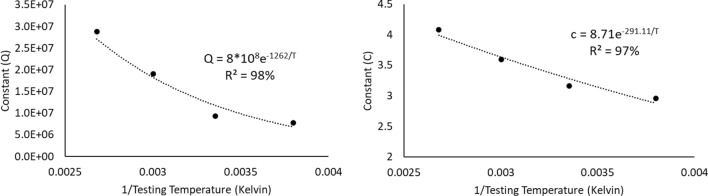
Modeling the evolutions in the stress life equation constants using the Arrhenius equation.

After performing the accelerated fatigue test on seven sample replicates (sample size) at different experimental conditions, the fatigue life data was collected, and the average values of the fatigue life were determined as shown in Table 2. A Two parameter Weibull distribution was utilized to describe the solder joint reliability. Figure 3 shows the probability plot of the Weibull distribution for SAC305 solder joints that were cycled at different stress amplitudes at room temperature (25 °C)^30^. A significant reduction in the solder joint life when the load level is increased can be obviously noted from the values of the characteristic life. The observed behavior of the fatigue life for the solder joints can be modeled by plotting the characteristic life values versus the cyclic stress amplitude. Then, the stress life equation was implemented as a fitting equation to illustrate this relationship, as depicted in Fig. 4. The R-squared value is used as a model adequacy metric to describe the ability of generated prediction models to estimate the desirable outcome values with high accuracy. To demonstrate the effect of changing the testing temperature on the solder joint’s reliability, the probability plots for the Weibull model were developed for the fatigue life data at different testing temperatures. Samples of the probability plots for the solder joints that are cycled at a − 10 °C testing temperature and different stress levels are displayed in Fig. 5^30^. Figure 6 represents the bar char illustrating a degradation in the characteristic life at different testing temperatures and stress amplitudes^30^. Figure 7 depicts the evolutions in the relationships between the stress level and fatigue life as the testing temperature changes. All the stress life equations that were implemented to predict the fatigue life at different conditions had high R-squared values (99%). A notable trend in the fatigue life equation constants can be observed in Fig. 7 when the testing temperature is changed. Table 3 displays the values of the stress life equation constants at different testing temperatures. The Arrhenius equation was applied to identify the relationships between the stress life equation constants values and the testing temperature in Kelvin scale by using the exponential function as shown in Fig. 8. The obtained equations from Fig. 8 were substituted in place of the fatigue life equation constants that are represented in Eq. (2) to construct a robust prediction equation (Eq. 6) of the fatigue life as a function of the testing temperature and stress amplitude. The obtained model adequacy value (R-squared) of Eq. (6) was 93%. In order to reflect the impacts of the experimental conditions on the solder joint reliability, the obtained prediction equation was used in the reliability equation model (Eq. 1) instead of the scale parameter. The relationship between the achieved shape parameter values and the experimental conditions was highly random, therefore, the mathematical average of the shape parameter was used as an estimator of the shape parameter value for the reliability model. Finally, Eq. (7) demonstrates a general reliability model for the SAC305 solder joint as a function of the fatigue life (cycles), stress level, and testing temperature.

6 $$N_{63} = 8*10^{8} *e^{ - 1262/T} P^{{ - 8.71*e^{ - 291.1/T} }}$$

7 $$R\left( t \right) = e^{{ - \left( {\frac{t}{{8*10^{8} *e^{ - 1262/T} P^{{ - 8.71*e^{ - 291.1/T} }} }}} \right)^{6.28} }}$$

**Table 2:** The average fatigue life at different experimental conditions.

Testing Temp. (°C)	Stress amplitude (MPa)	Average fatigue Life (Cycles)
− 10	16	1719
	20	726
	24	449
25	16	1318
	20	633
	24	378
60	16	352
	20	145
	24	84
100	16	134
	20	48
	24	17

**Table 3:** The stress life equation constants at different testing temperatures.

Testing temperature	Constant Q	Constant c
− 10	7.74*10^6^	2.955
25	9.32*10^6^	3.162
60	1.91*10^7^	3.593
100	2.88*10^7^	4.084

### The hysteresis loops behavior at different testing temperatures

**Figure 9:**
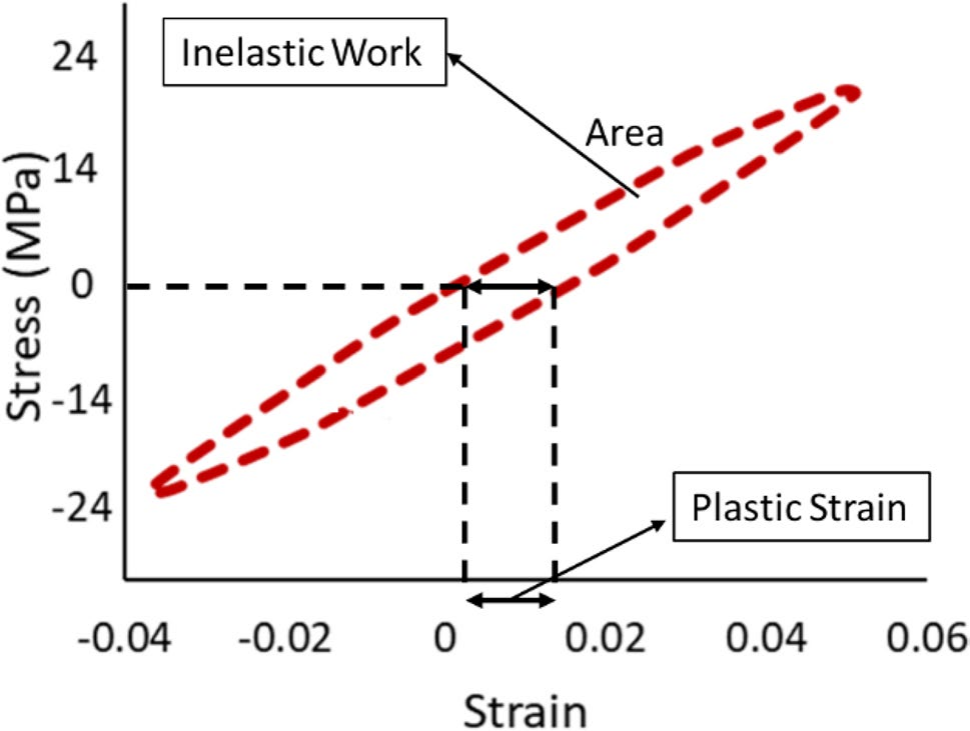
The hysteresis loop for the solder joint cycled at 24 MPa stress level and at 25 °C testing temperature.

**Figure 10:**
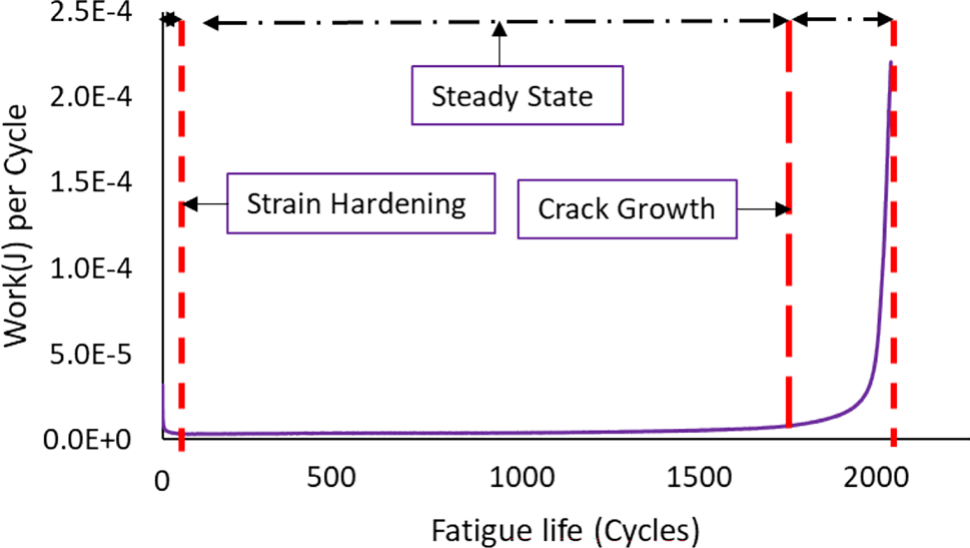
The evolutions in the inelastic work per cycle versus the fatigue life.

**Figure 11:**
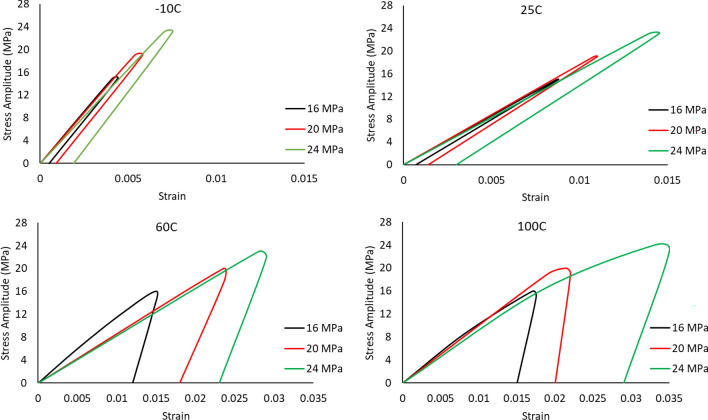
The evolutions in the hysteresis loop at different testing temperatures and stress amplitudes.

The hysteresis loop was developed for each cycle of the individual solder joints to demonstrate the evolutions in the fatigue properties of the solder joints at different experimental conditions. The inelastic work and plastic strain were determined from the constructed hysteresis loops. The value of the inelastic work was calculated by determining the area of the hysteresis loop and the shift in the strain at zero stress, which represents the plastic strain. The actual descriptions of the fatigue properties are the amount of work that was spent on each cycle and the amount of permanent deformation that was observed per cycle. Figure 9 illustrates the hysteresis loop for the solder joint cycled at a 24 MPa stress level and at a 25 °C testing temperature. Three main regions were defined in the solder joints life, which are strain hardening, steady state, and crack growth. The steady state region was specified for each tested individual solder joint by plotting the inelastic work or the plastic strain versus the cycle number of the solder joint. Figure 10 depicts the three regions of solder joint life for a solder joint tested at a -10 °C testing temperature and cycled at a 16 MPa stress amplitude. Then the evolutions in the hysteresis loop in the steady state region at different testing temperatures and load levels were identified, as shown in Fig. 11. An increase in the area of the hysteresis loop and the shift at stress zero were observed when either the testing temperature or the stress amplitude level were increased, as displayed in Fig. 11. Therefore, the inelastic work and plastic strain at steady state region were increased as well when the levels of the experimental conditions were increased. For the solder joints that were cycled at the same operating conditions, the average inelastic work and plastic strain per cycle in the steady state region were calculated.

### Reliability modeling using the fatigue properties

**Figure 12:**
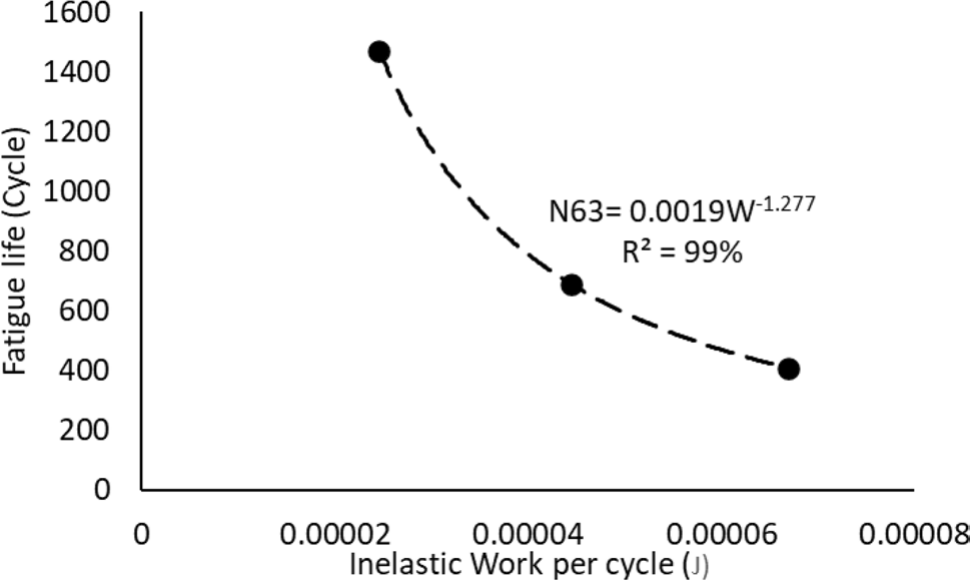
The relationship between the fatigue life and the average inelastic work per cycle for SAC305 solder joints that were examined at room temperature.

**Figure 13:**
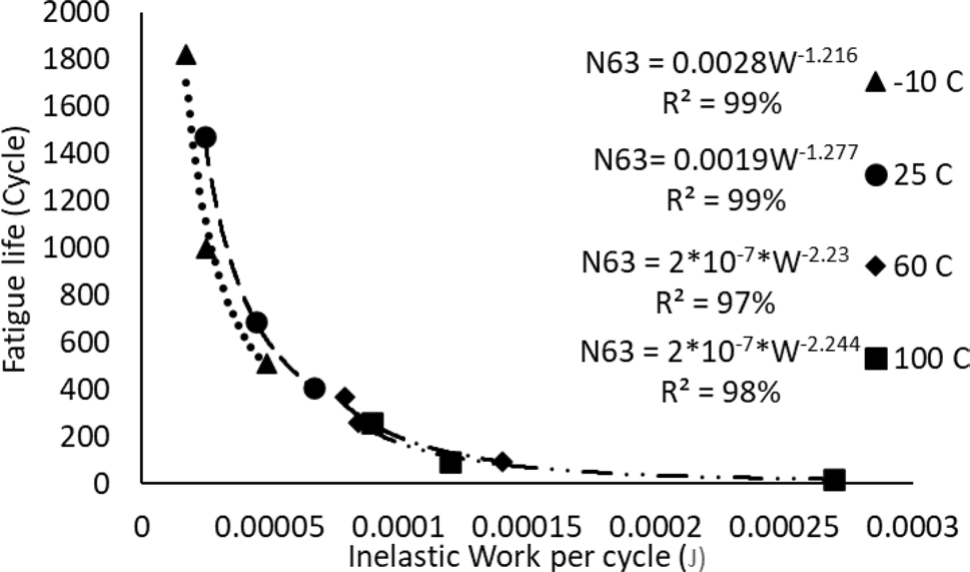
The impact of mutable testing temperature on the Morrow energy model.

**Figure 14:**
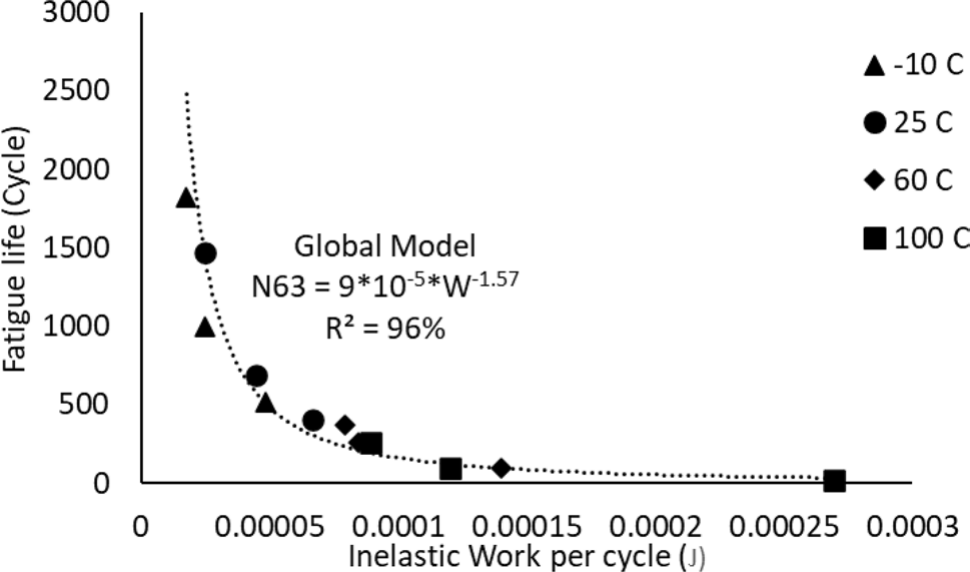
The Morrow energy global model.

The Morrow energy equation that is shown in Eq. (4) was utilized to model the relationship between the inelastic work and fatigue life of the solder joints. Figure 12 represents the Morrow energy model of the solder joints that were examined at different stress amplitudes when the testing temperature was fixed at 25 °C. The effect of changing the testing temperature on the Morrow equation constants is shown in Fig. 13. Table 4 represents the equation constants for the Morrow model that are determined from Fig. 13 by using Eq. (4). According to the results that were extracted from Fig. 13, the testing temperature didn’t significantly impact the Morrow energy model, therefore a global model was developed in Fig. 14 to predict the fatigue life as a function of inelastic work regardless of the fluctuations in the testing temperature values. The R-squared value of the proposed prediction equation was 96%. The global model parameters were defined in Table 4. The obtained equation from Fig. 14 was substituted in place of the scale parameter of the Weibull equation (Eq. 1). Equation (8) shows the final reliability model of SAC305 solder joints as a function of the inelastic work per cycle. The Morrow energy model was used, as mentioned before to predict the scale parameter of the Weibull model, and the shape parameter was estimated by determining the overall average of the shape parameter values at different conditions. Thus, the Morrow energy model represented a robust model against the change in the operating environmental temperatures.

8 $$R\left( t \right) = e^{{ - \left( {\frac{t}{{9*10^{5} *W^{ - 1.57} }}} \right)^{6.28} }}$$

**Table 4:** The morrow equation constants versus the testing temperature.

Testing temperature (°C)	Fatigue exponent (*Z*)	The ductility coefficient (*R*)
− 10	0.822368	0.007955
25	0.783085	0.007397
60	0.44843	0.000991
100	0.445633	0.001034
Global model	0.636943	0.001822

**Figure 15:**
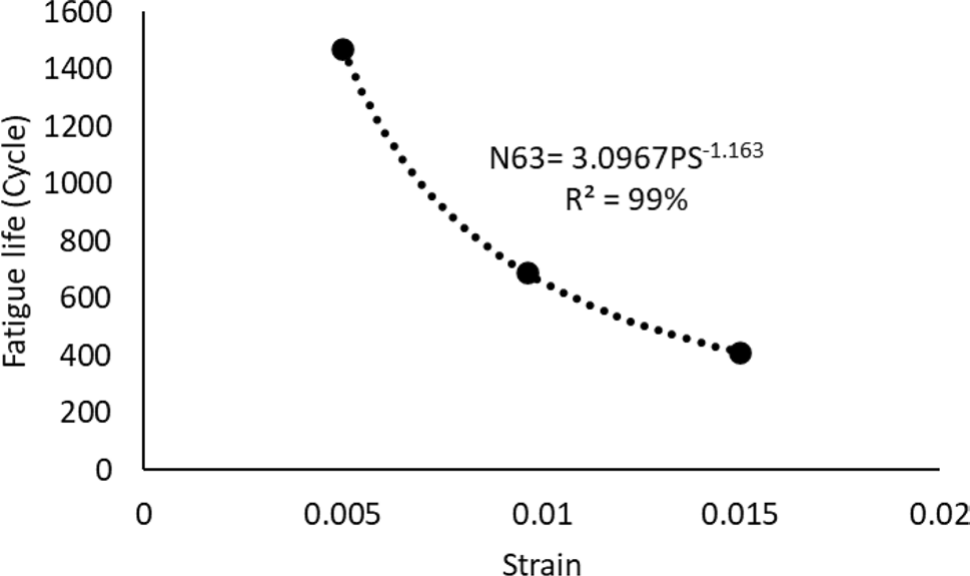
The Coffin Manson model of SAC305 solder that are cycled at room temperature and different stress levels.

**Figure 16:**
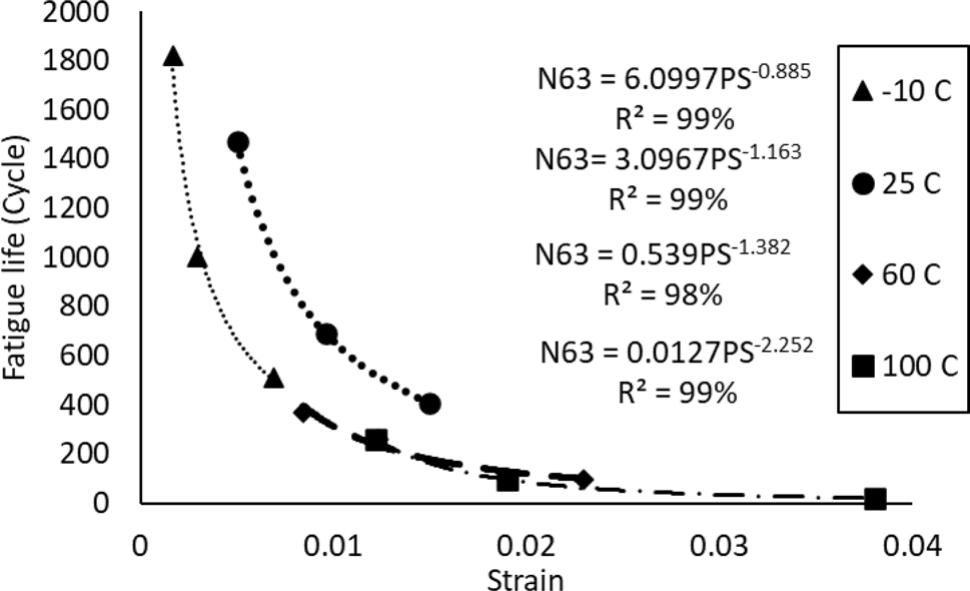
The Coffin Manson equation at different testing temperatures.

**Figure 17:**
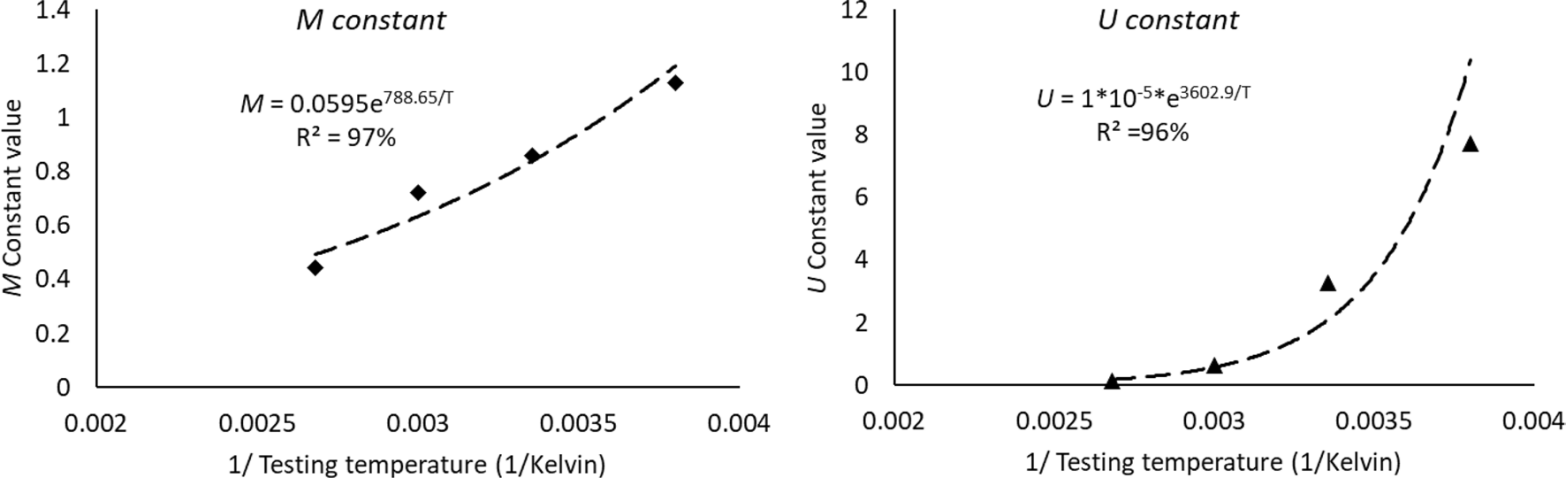
The prediction models for the coffin Manson parameters using the Arrhenious equation.

The power equation (Eq. 5) that represents the Coffin Manson model was used to quantify the relationship between the permanent deformation that was achieved from the applied stress which is called plastic strain, and the characteristic life. Figure 15 depicts the Coffin Manson equation for the solder joints that are cycled at room temperature and at different stress levels. A significant impact of the fluctuating testing temperature on the Coffin Manson model structure can be observed, as shown in Fig. 16. The Coffin Manson equation parameters (the fatigue exponent, and the ductility coefficient) can be driven from the equation constants that are displayed in Fig. 16. Table 5 represents the behavior of the fatigue exponent, and the ductility coefficient values at different stress levels. The mutable behavior of the Coffin Manson parameters at different testing temperatures were modeled by using the Arrhenius equation as shown in Fig. 17. The testing temperature was used in Kelvin scale when the Arrhenius model is applied. By utilizing the obtained equations from Fig. 17 in place of the Coffin Manson equation parameters provided in Eq. (5), a robust prediction model of the characteristic life as a function of the testing temperature and average plastic strain per cycle was formulated as depicted in Eq. (9). The R-squared value that represents the model adequacy for the obtained equation was 93%. A general reliability model of SAC305 solder joints using the Weibull distribution was constructed by using the prediction equation of the characteristic life that is shown in Eq. (9) to be substituted instead of the scale parameter of the Weibull distribution. Since, the shape parameter values were very random and the changing pattern of its values was unpredictable, the average value of the shape parameter at different experimental conditions was used as an estimator for the shape parameter value. The final reliability model is represented in Eq. (10).

9 $$N_{63} = \left( {1*10^{ - 5} *e^{{\frac{3602.9}{T}}} } \right)^{{\frac{1}{{0.0595*e^{788.65/T} }}}} PS^{{\frac{ - 1}{{0.0595*e^{788.65/T} }}}}$$

10 $$R\left( t \right) = e^{{ - \left( {\frac{t}{{\left( {1*10^{ - 5} *e^{{\frac{3602.9}{T}}} } \right)^{{\frac{1}{{0.0595*e^{788.65/T} }}}} PS^{{\frac{ - 1}{{0.0595*e^{788.65/T} }}}} }}} \right)^{6.28} }}$$

**Table 5:** The morrow equation constants versus the testing temperature.

Testing temperature (°C)	Fatigue exponent (*M*)	The ductility coefficient (*U*)
− 10	1.129944	7.715325
25	0.859845	3.270279
60	0.723589	0.639412
100	0.44405	0.143878

## Conclusion

This study examined the reliability of the individual SAC305 solder joints in actual operating settings at different experimental conditions. An accelerated fatigue shear test was utilized in the reliability assessment for the fatigue life of the solder joints. The stress amplitude and testing temperature with different factor levels were considered experimental parameters for the proposed test. A significant reduction in the fatigue life of the solder joints was observed when either the stress amplitude level or the testing temperature value was increased. The fatigue life behavior of the solder joint was identified at different operating conditions by using stress life and Arrhenius equations. The stress strain curve for the cycled solder joints was used to develop the hysteresis loops at different experimental conditions. Notable changes in the shape and magnitude of the developed hysteresis loops were found when the experimental parameters were varied. The averages of the inelastic work and the plastic strain per cycle at steady state region were extracted from the obtained hysteresis loops. Positive relationships were determined between the fatigue properties and the values of the testing temperature and load level. In contrast, the fatigue properties values were inversely proportional to the observed fatigue life. The Morrow energy and Coffin Manson models were utilized to define the relationships between fatigue life, plastic strain, and inelastic work. The Arrhenius model was implemented to describe the impacts of the mutable testing temperature environment on the structure of the Morrow energy and Coffin Manson models. Finally, three reliability models of the fatigue life were built based on the fatigue properties behavior and the applied stress amplitude and testing temperature.

## Data Availability

The datasets used and/or analyses during the current study available from the corresponding author on reasonable request.
