# Author Correction: Reliability modeling of the fatigue life of lead-free solder joints at different testing temperatures and load levels using the Arrhenius model

**DOI:** 10.1038/s41598-023-34175-y

**Published:** 2023-05-09

**Authors:** Dania Bani Hani, Raed Al Athamneh, Mohammed Abueed, Sa’d Hamasha

**Affiliations:** 1 Hijjawi Faculty for Engineering Technology, Yarmouk University, Irbid, 21163 Jordan; 2 Department of Industrial Engineering, Faculty of Engineering, The Hashemite University, P.O. BOX 330127, Zarqa, 13133 Jordan; 3 Intel Corporation, 2775 NE John Olsen Ave, Apt H116, Hillsboro, 97124 USA; 4 Department of Industrial and Systems Engineering Samuel Ginn College of Engineering, Auburn University, Auburn, AL 36849 USA

Correction to: *Scientific Reports* 10.1038/s41598-023-29636-3, published online 13 February 2023

The original version of this Article contained an error in the Reference list. Reference 30 was omitted and is listed below:

Al Athamneh, R., Abueed, M., Bani Hani, D., & Hamasha, S. D. Fuzzy Approach for Reliability Modeling of Lead-Free Solder Joints in Elevated Temperature Environmental Conditions. *Crystals, 12*(6), 775 (2022).

The original version of this Article was missing the information that the underlying dataset for this study has been previously published in [30]. The following paragraph has been added to the Material and Methods:

“Thus, the Arrhenius equation, stress life equation, Coffin Manson model and Morrow energy model were utilized in this study to construct three reliability models of SAC305 solder joints at different fatigue properties, working temperatures and load levels. The original data in this study was utilized before to model SAC305 solder joints fatigue life using different modeling methodologies and tools (Fuzzy inference system) [30], at which the proposed prediction models in the current study enhanced the predictability, simplicity, and accuracy of fatigue life modeling for SAC305 solder joints at different working temperatures and stress amplitudes.”

The legend of Figure 3 has been modified to reflect that the Figure was re-used from [30]. The updated legend for Figure 3 now reads:

“The Weibull probability plots for SAC305 solder joints cycled at room temperature and at different load levels (reused and modified from [30]).”

In the Results section under the subheading “Fatigue Failure Analysis Using Weibull Distribution” the following sentences were missing references to [30]:

“Figure 3 shows the probability plot of the Weibull distribution for SAC305 solder joints that were cycled at different stress amplitudes at room temperature (25°C).”

Now reads:

“Figure 3 shows the probability plot of the Weibull distribution for SAC305 solder joints that were cycled at different stress amplitudes at room temperature (25°C) [30]. “

“Samples of the probability plots for the solder joints that are cycled at a -10°C testing temperature and different stress levels are displayed in figure 5.”

Now reads:

“Samples of the probability plots for the solder joints that are cycled at a -10°C testing temperature and different stress levels are displayed in figure 5 [30].”

“Figure 6 represents the bar char illustrating a degradation in the characteristic life at different testing temperatures and stress amplitudes.”

Now reads:

“Figure 6 represents the bar char illustrating a degradation in the characteristic life at different testing temperatures and stress amplitudes [30].”

The original article has been updated.

